# Differences in the Level of Electronic Health Literacy Between Users and Nonusers of Digital Health Services: An Exploratory Survey of a Group of Medical Outpatients

**DOI:** 10.2196/ijmr.8423

**Published:** 2019-04-05

**Authors:** Kamila Adellund Holt, Astrid Karnoe, Dorthe Overgaard, Sidse Edith Nielsen, Lars Kayser, Michael Einar Røder, Gustav From

**Affiliations:** 1 Department of Nursing Faculty of Health University College Copenhagen Copenhagen N Denmark; 2 Section of Social Medicine Department of Public Health University of Copenhagen Copenhagen Denmark; 3 Danish Multiple Sclerosis Society Copenhagen Denmark; 4 Medical Department Herlev-Gentofte University Hospital Copenhagen Denmark; 5 Steno Diabetes Center Odense Odense University Hospital Odense Denmark

**Keywords:** health literacy, computer literacy, questionnaires, telemedicine, consumer health informatics

## Abstract

**Background:**

Digitalization of health services ensures greater availability of services and improved contact to health professionals. To ensure high user adoption rates, we need to understand the indicators of use and nonuse. Traditionally, these have included classic sociodemographic variables such as age, sex, and educational level. Electronic health literacy (eHL) describes knowledge, skills, and experiences in the interaction with digital health services and technology. With our recent introduction of 2 new multidimensional instruments to measure eHL, the eHL questionnaire (eHLQ) and the eHL assessment (eHLA) toolkit, eHL provides a multifaceted approach to understand use and nonuse of digital health solutions in detail.

**Objective:**

The aim of this study was to investigate how users and nonusers of digital services differ with respect to eHL, in a group of patients with regular contact to a hospital outpatient clinic. Furthermore, to examine how usage and nonusage, and eHL levels are associated with factors such as age, sex, educational level, and self-rated health.

**Methods:**

Outpatients were asked to fill out a survey comprising items about usage of digital services, including digital contact to general practitioner (GP) and communication via the national health portal sundhed.dk, the eHLQ, and the eHLA toolkit, as well as items on age, sex, education, and self-rated health. In total, 246 patients completed the survey. A Mann-Whitney test was used to test for differences between users and nonusers of digital services. Correlation tests described correlations between eHL scales (eHEALSs) and age, education, and self-rated health. A significance level of .0071 was used to reject the null hypothesis in relation to the eHEALSs and usage of digital services.

**Results:**

In total, 95.1% (234/246) of the participants used their personal digital ID (NemID), 57.7% (142/246) were in contact with their GPs electronically, and 54.0% (133/246) had used the national health portal (sundhed.dk) within the last 3 months. There were no differences between users and nonusers of sundhed.dk with respect to age, sex, educational level, and self-rated health. Users of NemID scored higher than nonusers in 6 of the 7 dimensions of eHLQ, the only one which did not differ was dimension 2: *Understanding of health concepts and language.* Sundhed.dk users had a higher score in all of the 7 dimensions except for dimension 4: *Feel safe and in control*. The eHLA toolkit showed that users of sundhed.dk and NemID had higher levels of eHL with regard to tools 2, 5, 6, and 7. Furthermore, users of sundhed.dk had higher levels of eHL with regard to tools 3 and 4.

**Conclusions:**

Information about patients’ eHL may provide clinicians an understanding of patients’ reasons for not using digital health services, better than sociodemographic data or self-rated health.

## Introduction

### Background

The ongoing extensive digitalization of health services worldwide may be considered an advantage for many people, as the use of information and communications technology (ICT) ensures greater availability of services and better contact to health service professionals [1].

In line with this development, in Denmark, public services are highly digitalized, and citizens communicate with public authorities via a digital portal. This includes electronic communication via a national email service called patient’s digital mailbox (e-Boks) [2]. Only people who are not able to access computers or use the digital services can be exempted from this (currently 8.9%) [2]. Digital access to all public services is governed by the national identification system NemID. In total, 98% of the population above 15 years has access to NemID [3]. Since 2009, the national health portal sundhed.dk has facilitated citizens’ access to the national, regional, and local health care services, their communication with health professionals, and their access to health-related information [4]. The access to nonperson-specific information about health services and the health-related information is publicly available, but access communication that includes personal data, for example, clinical data and communication with one’s general practitioner (GP) for renewal of drug prescriptions, requires a personal log-in using the NemID log-on. Public authorities communicate with citizens via e-Boks to send information letters from hospitals, including the outpatient clinic referrals.

This increasing tendency toward mandatory digital communication with public services, including health care services, as is seen in Denmark, calls for attention to a problem: how can we include citizens who are not able to take advantage of the new digital opportunities and obtain the full benefits of digitalization?

Previously, reasons for not using digital services or technologies were considered to be that users lack access to, have resistance to, or reject using the digital services as they do not find it beneficial [5].

This simplified view has been challenged in recent years by studies that explore, in detail, the reasons for not using digital health services. A recent review identified several key barriers to successful adoption of digital health interventions [6]. These barriers were related to both personal attributes such as agency, motivation, personal life experiences, and values, and the context, that is, the health care providers’ approach to engaging and recruiting users, as well as the quality of the solution. In alignment with this, a Danish study, which examined why older people (aged above 58 years) use public digital services, identified that motivation, convenience, and skills were important factors for adopting digital solutions [7]. Two recent qualitative studies support this finding and also show that the patients’ context and condition may also influence their preferences. The first of the 2 qualitative studies is a Danish study based on 10 patients with more than 1 chronic condition (multimorbid patients), which argues that patients’ motivation to use ICT is positively related to the burden of their disease [8]. The other study from the Netherlands, including 17 patients with a chronic heart disease, reveals several other important factors for patients choosing to use an electronic health (eHealth) portal: a more personal contact to a coach, self-perceived computer skills, and factors related to how the platform is introduced and used [9].

In a newly published study with 201 informants examining the willingness to use eHealth portals, authors found that the willingness to use a health portal was related to younger age, higher self-rated health, an education level above high-school level, higher acculturation status, higher computer literacy, and adequate health literacy [10].

All these studies point to a complexity of reasons for adoption and use of digital health services, of which many relate to the individual’s knowledge, skills, perception, and experiences, including trust and motivation, but also relate to the way health professionals introduce new technologies [11].

### Electronic Health Literacy

In 2015, Monkman and Kushniruk proposed a new model of Consumer Health Information System adoption [12]. In this model, they combine an understanding of the usability of the products with the users’ eHealth literacy (eHL)—a model which is in alignment with the suggestion by Kayser et al that it may be important to address users’ eHL to achieve design solutions that suit the user’s needs and capabilities better [13]. This calls for further investigation of the role of eHL as a means to understand the adoption and usage of digital health services in the context of the user interacting with the services and technology.

The original concept of eHL or digital health literacy was introduced in 2006 by Normann and Skinner [14], and it highlights the users’ competence needed to engage with digital health services. Using the Normann and Skinner model and the related instrument, eHL scale (eHEALS), a positive correlation between information-seeking behavior and eHL has been demonstrated, for example, in 31 patients with rheumatoid arthritis in the Netherlands [15], 2371 parents of children with severe conditions in the United States [16], and in several thousand consumers in Israel [17]. The latter also reported that there was no relation between self-reported health and eHL [17].

eHL has also been shown to correlate positively with the users’ educational level but correlate negatively to age [15,18].

Although these studies have linked eHL to digital behavior, their findings were mainly based on the eHEALS instrument that directly evaluates information-seeking behavior on the internet in relation to health.

Even though eHEALS is still a widely used tool [19-22], it does not provide sufficient understanding of the individual’s interaction with digital services and technology. In 2011, van der Vaart [15] already called for the need of a new understanding of eHL after the internet had been turned into a more dynamic Web 2.0 media. Moreover, in 2017, Griebel et al pointed to the need of new ways to describe eHL with a broader view on the digital health consumer perspective [23].

With the development of 2 new tools, we have introduced a new understanding of people’s eHL, including knowledge, skills, perceptions, and experiences in relation to their usage of digital health services and health technology. One measure, the eHL questionnaire (eHLQ), is developed as an instrument to access the 7 dimensions of the eHL framework (eHLF), which describes users’ knowledge, skills, perception, and experiences in relation to digital health services and health technologies [24].

The 35 items of eHLQ emerged from a condensation of more than 450 statements that constituted the fundament for the development of the eHLF. In this way, the final items capture a somewhat higher-order assessment of the respondent’s understanding and engagement in health information, which is more suitable for the intended usage as a psychometrically sound and valid instrument and is not intended to act as an inventory [24].

The other instrument, the eHL assessment (eHLA) toolkit, examines eHL by combining specific elements from health literacy and digital literacy as both self-reported and performance tests [25].

### Objectives

With this new multifaceted approach, we are able to contribute to a better understanding of how users differ from nonusers of digital services, not only with respect to personal attributes such as age, sex, educational level, and self-rated health but also with a particular focus on the individuals’ knowledge, skills, perception, and experiences with digital health services.

Consequently, our research question is how can a multifaceted evaluation of individuals’ eHL be used to understand usage and nonusage of digital health services and how are usage and eHL related to age, sex, educational level, and self-rated health?

## Methods

### Study Design

We used a quantitative cross-sectional study design, collecting data using Danish versions of the eHLQ and eHLA instruments, both validated in a Danish population. In total, 246 patients diagnosed with diabetes, other endocrine conditions, and/or gastrointestinal diseases were included. The patients were consecutively enrolled when visiting the outpatient clinic at the Gentofte Hospital, Denmark, between November 2015 and March 2016.

**Figure 1:**
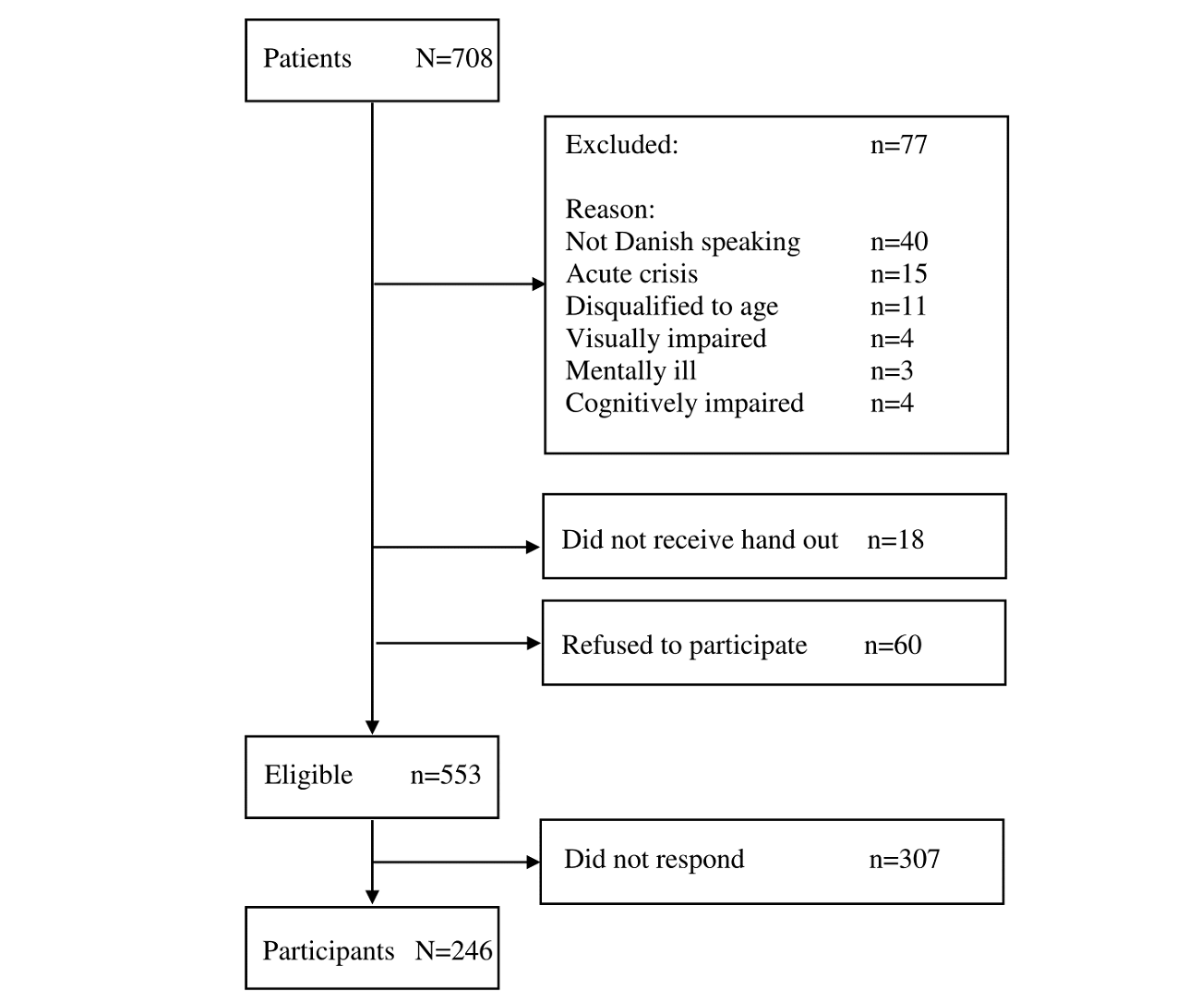
Flowchart for inclusion of patients in the study.

Patients were excluded if they were under the age of 18, had insufficient cognitive functions, or did not understand Danish. The distribution of the questionnaire containing the 2 instruments was undertaken by the nurses at the outpatient clinic, who also assessed whether the respondent had sufficient cognitive functions to participate. In some cases, the nurses also judged from an ethical perspective that the patients, for reasons not stated in the protocol, should be excused from participation in the study (see Figure 1). Patients either filled out the questionnaire in the waiting room or completed it at home and returned it in a prepaid envelope. Patients did not receive reminders.

A total of 553 patients were given the questionnaire to complete. Of these, 246 filled in and returned the questionnaire, whereas 307 did not respond, resulting in a response rate of 44.4% (246/553; Figure 1).

### Questionnaires

The questionnaire battery contained eHLQ, eHLA, and questions concerning the patient’s sociodemographics, digital behavior, and self-rated health.

The questionnaire battery also included questions about whether informants were exempted from using e-Boks, had used their NemID within the previous 6 months, and whether they had logged into sundhed.dk within the previous 3 months. Finally, questions about the informants’ communication with their GP were included.

### Educational Level

The demographic variable education was aggregated to 4 levels:

1. *Comprehensive school* equivalent to International Standard Classification of Education 2011 (ISCED-2011) levels 1 and 2 or European Qualifications Framework (EQF) level 2.
2. Short education equivalent to ISCED and EQF levels 3, 4, and 5.
3. Medium education equivalent to ISCED and EQF level 6.
4. Long education equivalent to ISCED and EQF levels 7 and 8 [26,27].

### Self-Rated Health

The levels reported in eHLQ and eHLA were correlated to self-rated health, which was measured on a 5-point Likert scale from excellent to poor [28,29]. For the statistical evaluation, the scale was reversed so that *excellent health* was given the highest score (5) and *poor health* the lowest score (1).

### Sociodemographic Data

The participants’ sociodemographic characteristics are provided in Table 1. The mean age was 56.5 with a range from 18 to 89 years.

### Electronic Health Literacy Questionnaire

eHLQ is a validated and psychometrically sound instrument that comprises 35 items covering 7 dimensions: (1) *using technology to process health information*, (2) *understanding of health concepts and language*, (3) *ability to actively engage with digital services*, (4) *feel safe and in control*, (5) *motivated to engage with digital services*, (6) *access to digital services that work*, and (7) *digital services that suit individual needs* [30]. Dimensions 1 and 2 describe the patient’s individual competence, dimensions 3 to 5 describe the interaction between the patient and the digital services, and dimensions 6 and 7 characterize the patient’s experience with digital systems or services. Each of the first 5 dimensions contains 5 items, whereas dimension 6 has 6 items and dimension 7 has 4 items. Each item has 4 options, *strongly disagree*, *disagree*, *agree*, and *strongly agree*, which yield 1 to 4 points, respectively.

### Electronic Health Literacy Assessment Toolkit

eHLA is a validated and psychometrically sound instrument that contains 4 (1-4) health literacy tools and 3 (5-7) digital literacy tools. The tools describe (1) *functional health literacy*, (2) *self-assessed health literacy*, (3) *familiarity with health and disease*, (4) *knowledge of health and disease*, (5) *digital familiarity*, (6) *digital confidence*, and (7) *digital incentives* [25].

The eHLA comprises 44 items: 10 items in tool 1 (*functional health literacy*), 9 items in tool 2 (*self-assessed health literacy)*, 5 items in tool 3 (*familiarity with health and disease*), 6 items in tool 4 (*knowledge of health and disease*), 6 items in tool 5 (*digital familiarity*), 4 items in tool 6 (*digital confidence*), and 4 items in tool 7 (*digital incentives*). Tools 1 and 4 are performance tests. In tool 1, *functional health literacy*, 1 point is given for each correct answer, and in tool 4, *knowledge of health and disease*, 2 points are given for each correct answer and 1 point for opting out. The remaining 5 tools have a 4-option scale. In tool 2, the scale ranges from *very difficult* to *very easy*, in tool 3, the score ranges from *no* *knowledge* to *full knowledge*, in tool 5, from *not* *at all familiar* to *completely familiar*, and in tool 6, from *very unconfident* to *very confident*. The items in tool 7 are assessed on a scale ranging from *strongly disagree* to *strongly agree*.

**Table 1:** The distribution of sociodemographics and self-rated health.

Total		Statistics (N=246), n (%)
**Sex**		
	Female	137 (55.7)
	Male	109 (44.3)
**Education**		
	Comprehensive school	19 (7.2)
	Short education	70 (28.5)
	Medium education	84 (34.1)
	Long education	65 (26.4)
**Self-rated health**		
	Poor	8 (3.3)
	Less well	61 (24.8)
	Well	108 (43.9)
	Extremely well	59 (23.9)
	Excellent	9 (3.7)
**Patients’ condition**		
	Diabetes	92 (37.4)
	Other	154 (62.6)

### Statistical Analyses

Descriptive statistics are reported as means and interquartile range for age, educational level, and self-rated health. Differences in scores between male and female and users and nonusers of sundhed.dk and NemID were tested using the nonparametric Mann-Whitney test. Differences between users and nonusers of sundhed.dk and NemID with respect to sex were tested using Pearson Chi-square test. We tested for correlation among eHL and age, educational level, and self-rated health. We interpreted the strength of the correlation in accordance with Brace (weak ≤±.2, ±.3 to .6 moderate, strong ≥±.7) [31]. A Bonferroni correction was made for univariate analyses for each of the 2 tools relating sociodemographic factors to each of the tools’ 7 dimensions (alpha/number of hypotheses→.05/7=.0071) [32].

### Ethics

The research complied with the Helsinki declaration, and the study was approved by the Danish Data Protection Agency (2012-58-004 under the capital Region of Denmark local record number HGH-2018-021 I-suite 06245). Information about the survey was given to the patients in accordance with the inclusion criterion, and written informed consent was obtained beforehand from all the participants.

## Results

### Use of Digital Services Among Outpatients

The results showed that 142/246 (57.7%) of the outpatients were in contact with their GP via email or econsultation on the GP’s website. The use of NemID was widespread: 234/246 (95.1%) patients had used it in the previous 6 months to communicate with public authorities, access home banking or a Web portal for citizens.

**Figure 2:**
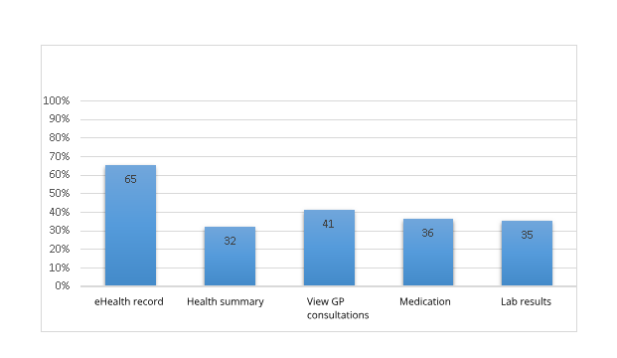
The participants’ (N=133) use of functionalities on sundhed.dk. eHealth: electronic health; GP: general practitioner.

A total of 133/246 (54.1%) patients had visited sundhed.dk within the previous 3 months; the majority of patients used the website to access their own eHealth record (Figure 2).

There was no difference between males and females (*P*=.87) age (*P*=.22), self-rated health (*P*=.09), or educational level (*P*=.29) between users and nonusers of sundhed.dk. Users of NemID had a lower mean age 56 (45-68) years versus 67 (54-82) years (*P=*.01) and higher score of self-rated health, 3.0 (2-4) versus 2.2 (2-3) (*P*=.001), than nonusers. There was no difference in educational level between users and nonusers of NemID (*P*=.14), and there were no differences in usage between males and females (*P*=.68).

Percentages in Figure 2 are calculated on the basis of 133 users of sundhed.dk.

In total, 202 patients of the 234 patients with nemID (86.3%) had activated the functionality of receiving an email notification when an official institution sent a letter to the e-Boks. Only 6.4% (15/234) patients were assisted by friends or family in the use of different features in the digital mailbox. A total of 4.5% (11/234) patients were exempted from using the mandatory digital mailbox.

In dimensions 1, 3, 5, 6, and 7, scores of eHLQ dimensions were higher for users than for nonusers for both NemID and sundhed.dk. In dimension 2, *understanding of health concepts and language*, users of sundhed.dk but not users of NemID scored significantly higher, and in dimension 4, *feeling safe and in control*, only NemID users but not sundhed.dk users scored higher than nonusers (Table 2).

Results from the eHLA toolkit showed that users of sundhed.dk and NemID scored significantly higher in tools 2 (*self-assessed health literacy*), 5 (*digital familiarity*), and 7 (*digital incentives*). In addition, users of sundhed.dk also scored significantly better in tool 3 (*familiarity with health and disease*), and users of NemID scored significantly better in tool 6 (*digital confidence*; Table 3).

**Table 2:** Differences in the 7 dimensions in electronic health literacy questionnaire between users and nonusers of sundhed.dk and NemID.

Dimension name in eHLQ^a^	Mean	Users of sundhed.dk			Users of NemID		
		Yes/No, N	Mean (IQR^b^)	*P* value	Yes/No, N	Mean (IQR)	*P* value
1. *Using technology to process health information*	2.7	Yes, 132	2.9 (2.6-3.4)	<.001	Yes, 230	2.8 (2.4-3.2)	<.001
		No, 94	2.5 (2.2-3.0)		No, 8	1.5 (1.0-2.0)	
2. *Understanding of health concepts and language*	3.1	Yes, 132	3.2 (3.0-3.6)	.004	Yes, 230	3.2 (2.8-3.4)	.56
		No, 94	3.0 (2.6-3.4)		No, 8	2.9 (1.9-3.8)	
3. *Ability to actively engage with digital services*	3.0	Yes, 132	3.1 (2.8-3.6)	<.001	Yes, 230	3.0 (2.6-3.6)	<.001
		No, 94	2.8 (2.4-3.4)		No, 8	1.6 (1.0-2.0)	
4. *Feel safe and in control*	2.8	Yes, 131	2.9 (2.6-3.2)	.58	Yes, 228	2.9 (2.6-3.2)	.003
		No, 93	2.8 (2.5-3.2)		No, 8	2.1 (1.6-2.6)	
5. *Motivated to engage with digital services*	2.7	Yes, 131	2.9 (2.6-3.4)	<.001	Yes, 229	2.8 (2.4-3.2)	<.001
		No, 95	2.5 (2.0-3.0)		No, 9	1.6 (1.2-2.0)	
6. *Access to digital services that work*	2.7	Yes, 133	2.8 (2.3-3.0)	.007	Yes, 231	2.7 (2.3-3.0)	.001
		No, 95	2.6 (2.2-3.0)		No, 9	2.0 (1.7-2.5)	
7. *Digital services that suit individual needs*	2.6	Yes, 130	2.7 (2.3-3.0)	.005	Yes, 226	2.6 (2.3-3.0)	.001
		No, 93	2.4 (2.0-3.0)		No, 9	1.7 (1.0-2.0)	

^a^eHLQ: eHealth literacy questionnaire.

^b^IQR: interquartile range.

**Table 3:** Differences in the 7 tools in electronic health literacy assessment between users and nonusers of sundhed.dk and NemID.

Tool in eHLA^a^	Mean	Users of sundhed.dk			Users of NemID		
		Yes/No, N	Mean (IQR^b^)	*P* value	Yes/No, N	Mean (IQR)	*P* value
1. Functional health literacy	9.5	Yes, 120	9.5 (9-10)	.75	Yes, 207	9.5 (9-10)	.89
		No, 86	9.4 (9-10)		No, 8	9.5 (9-10)	
2. Self-assessed health literacy	3.3	Yes, 123	3.3 (2.9-3.7)	.004	Yes, 217	3.2 (2.9-3.6)	.007
		No, 93	3.1 (2.8-3.3)		No, 11	2.8 (2.6-3.2)	
3. Familiarity with health and disease	3.1	Yes, 124	3.3 (3.0-3.8)	.006	Yes, 221	3.1 (2.6-3.8)	.58
		No, 95	2.9 (2.4-3.8)		No, 10	3.0 (2.8-3.4)	
4. Knowledge of health and disease	9.7	Yes, 119	9.9 (9-12)	.01	Yes, 213	9.6 (8-11)	.41
		No, 92	9.3 (8-10)		No, 10	10.1 (8-12)	
5. Digital familiarity	3.5	Yes, 125	3.7 (3.5-4.0)	<.001	Yes, 220	3.6 (3.5-4.0)	<.001
		No, 95	3.2 (2.8-4.0)		No, 12	1.7 (1.0-2.3)	
6. Digital confidence	3.4	Yes, 129	3.6 (3.3-4.0)	.02	Yes, 225	3.5 (3.3-4.0)	<.001
		No, 92	3.3 (2.8-4.0)		No, 8	1.7 (1.0-2.3)	
7. Digital incentives	3.5	Yes, 133	3.6 (3.3-4.0)	.005	Yes, 230	3.5 (3.0-4.0)	<.001
		No, 93	3.3 (2.8-4.0)		No, 8	2.0 (1.3-2.8)	

^a^eHLA: eHealth literacy assessment.

^b^IQR: interquartile range.

### Relation Among Electronic Health Literacy Questionnaire, Electronic Health Literacy Assessment and Age, Sex, Education, and Self-Rated Health

Age is weakly and negatively correlated to eHLQ dimension 3 (*ability to actively engage with digital services*; Table 4). Using the Mann-Whitney test, we did not find any differences between the eHLQ scores for males and females. Educational level was weakly and negatively correlated to dimensions 4 (*feel safe and in control*) and 6 (*access to digital services that work*; Table 4).

Patients’ self-rated health showed a positive, weak correlation with 4 of the 7 dimensions: 1 (*using technology to process health technologies*), 3 (*ability to actively engage with digital services*) 5 (*motivated to engage with digital services*) and 6 (*access to digital services that work*).

Three of the 7 eHLA tools were associated with age: tool 6 *(digital confidence* was moderate, negative correlate). Tools 5 (*digital familiarity*) and 7 (*digital incentives*) showed a weak negative correlation. Educational level was weakly and positively correlated with tools 4 (*knowledge of health and disease*) and 5 (*digital familiarity*). Self-rated health was weakly and positively correlated with 2 (*self-assessed health literacy*), 5 (*digital familiarity*), 6 (*digital confidence*), and 7 (*digital incentives;* Table 5). The Mann-Whitney test for differences between sexes revealed a significantly higher score for males than females in tool 5 (*digital familiarity*, *P*=.005).

**Table 4:** The correlations among dimensions for electronic health literacy questionnaire and age, education, and self-rated health.

Dimension name in eHLQ^a^	Age		Education		Self-rated health^b^	
	Coefficient	*P* value	Coefficient	*P* value	Coefficient	*P* value
1. *Using technology to process health information*	−.12	.01	0.09	.08	0.16	.002
2. *Understanding of health concepts and language*	−.02	.61	0.14	.01	0.1	.05
3. *Ability to actively engage with digital services*	−.23	<.001	0.09	.08	0.18	.001
4. *Feel safe and in control*	−.01	.8	−.17	.002	0.08	.12
5. *Motivated to engage with digital services*	−.10	.03	0.04	.46	0.23	<.001
6. *Access to digital services that work*	−.07	.1	−.14	.005	0.14	.005
7. *Digital services that suit individual needs*	−.09	.05	−.08	.13	0.13	.01

^a^eHLQ: eHealth literacy questionnaire. ^b^Self-rated health: 1=poor health, 5=excellent health.

**Table 5:** The correlation between tools in electronic health literacy assessment and age, education, and self-rated health.

Tool in eHLA^a^	Age		Education		Self-rated health^b^	
	Coefficient	*P* value	Coefficient	*P* value	Coefficient	*P* value
1. Functional health literacy	−.08	.13	.16	.01	.05	.45
2. Self-assessed health literacy	−.05	.31	<.001	.95	.18	.001
3. Familiarity with health and disease	.08	.09	.11	.05	.03	.62
4. Knowledge of health and disease	.11	.02	.15	.007	.03	.60
5. Digital familiarity	−.25	<.001	.22	<.001	.25	<.001
6. Digital confidence	−.34	<.001	.13	.02	.20	<.001
7. Digital incentives	−.17	<.001	.11	.04	.20	<.001

^a^eHLA: eHealth literacy assessment. ^b^Self-rated health: 1=poor health, 5=excellent health.

## Discussion

The introduction of the 2 new, recently validated multidimensional measures of eHL, eHLQ and eHLA toolkit, allows us to examine patients’ digital behavior from a multifaceted approach, offering a better understanding of whether knowledge, skills, perception, or experiences are related to usage of digital services. As described in the following, this offers a richer understanding than just judging the users’ capabilities on the basis of their sociodemographic data such as age, sex, educational level, and self-rated health.

### Usage of Digital Services

The lack of a difference with respect to age and sex between the users and nonusers of the digital health service sundhed.dk corresponds with the findings of Siren and Stellefson [7,33].

We did find a difference in age between users and nonusers of the public digital service NemID. This may be explained by the relatively high adoption of NemID in the Danish society; consequently, those not using NemID are mainly excluded because of high age and disabilities. The latter is also supported by the finding that nonusers also had a lower score of self-reported health. Whether this relatively small but vulnerable group of 5% can benefit from digital inclusion remains to be investigated, but the health professionals should be aware of this particular group.

Here, multidimensional measures such as eHLQ and eHLA can add to our understanding of areas that might need to be addressed, as discussed in the following.

Although 95% of the participants in the study use NemID to access digital services, less than 60% of the participants have been in contact with their GP electronically or have taken advantage of the functions available on sundhed.dk. This could be explained by the fact that some NemID services, for example, the electronic mailbox for communications from public authorities, are mandatory to use, whereas electronic contact to one’s GP and use of the portal sundhed.dk are voluntary.

The conflicting results in the literature regarding the association among eHealth usage and users’ age, sex, or educational level may be explained by the context and research question. A study where the actual usage is reported as in this study may differ from studies where the focus is on, for example, the users’ willingness to use a health portal or a medical record, such as reported by Trubitt et al, which found an association with age and education. This is in contrast to our finding that there were no differences between users and nonusers of sundhed.dk with respect to age and education [10].

### Electronic Health Literacy and Usage of Digital Services

In general, users of NemID and sundhed.dk scored higher in most dimensions of the eHLQ. Moreover, users tended to score higher in 4 of the 7 eHLA tools for both sundhed.dk (tools 2, 3, 5, and 7) and NemID (tools 2, 5, 6, and 7). It should be noticed that sundhed.dk users had a higher score in the eHLQ dimension 2 as well as 2 of the 4 health literacy tools in eHLA, whereas the 2 other tools in eHLA that related to functional tests did not differ. This suggests that the users of sundhed.dk, because of their better understanding of the health-related language and concepts, are better equipped to understand the information and interact with the services in the health portal.

The assumption that health literacy is a determinant of the usage of the digital health services can be supported by the finding that NemID users did not differ from nonusers with respect to eHLQ dimension 2 and the eHLA tools 1, 3, and 4, which indicates that usage of other public digital services is not related to the users’ health literacy.

It is noteworthy that we did not find any differences in scores for the eHLQ dimension 4 (*feeling safe and in control*) between users and nonusers of sundhed.dk. This contrasts with our findings for users of NemID; here, a difference was found. An interpretation of this finding could be that although trust has no significance for the decision to use eHealth technologies such as the health portal sundhed.dk, the relatively few nonusers of NemID may have concerns about safety because of a lack of insight into how the services function.

Our finding is in accordance with Siren and Knudsen [7], who also found that the feeling of being safe and in control is not in itself a significant factor for using digital health services. It can be speculated whether being a patient in the health care sector makes people more trusting when they access and use health services.

### Electronic Health Literacy in Relation to Sociodemographic Data

The eHL level only differed between males and females with respect to 2 of the eHLA tools, but it did not differ in any of the dimensions in the eHLQ.

It is of particular interest that the digital tool 5 (*digital familiarity*) in the eHLA toolkit showed a higher score in males compared with females. This is in accordance with the findings of Hargittai et al, who measured digital literacy in college students using a scale that has inspired the construction of this tool [34].

Scales of eHLQ and eHLA that relate to digital skills showed significant negative correlations with age. This may reflect that people of older age generally have more problems finding information, which may contribute to less motivation to engage with technology [15]. Moreover, an increased need of health services by elder individuals may give rise to a feeling that the services are not sufficiently suited to their needs. This was reflected in a negative correlation between age and eHLQ dimension 7 (*access to digital services that suit individual needs*; *P*=.05); however, it was not significant after a correction for multiple comparisons using Bonferroni.

For the eHLQ dimensions, educational level was weakly, negatively correlated with dimension 4 (*feel safe and in control*) and dimension 6 (*access to digital services that work*). The negative correlation in dimensions 4 *(feel safe and in control*) and 6 *(access to digital services that work*) may be because of a general skepticism toward digital services. The finding that people with a higher educational level tend to have less trust is in alignment with a recent study from a European Union (EU) project evaluating an eHealth solution, the Health Monitor, with both patients and health professionals. In this study, health professionals tended to have more concerns about data privacy than lay people [18].

In contrast to the negative correlation between the 2 eHLQ dimensions and 2 of the eHLA tools; tool 4 (*knowledge of health and disease*) and tool 5 (*digital familiarity*) were positively correlated with the educational level. This finding is inconclusive, as several of the other tools within both health literacy and digital literacy are not related to educational level.

Although studies based upon eHEALS point to an association among eHL and age and educational level [15-17,35], our data are, apart from the negative correlation between age and the digital scales, not conclusive. Combined with our finding, that age and educational level do not differ between users and nonusers of sundhed.dk, this suggests that other factors may contribute to the adoption of eHealth service usage.

### Electronic Health Literacy and Self-Rated Health

Most interestingly, eHLA’s tool 2, which was derived from the European Health Literacy Survey HLS-EU, had a positive correlation with self-rated health similar to earlier reports in relation to the full HLS-EU instrument [36]. As self-rated health is often positively associated with health literacy [37], it would be expected that those tools and dimensions that are related to health literacy would also be positively correlated with self-rated health. Interestingly, eHLQ dimension 2 (*understanding health concepts and language*) as well as 3 of the 4 health literacy tools (1, 3, and 4) in eHLA did not exhibit such a correlation. However, 4 of the eHLQ scales as well as the 3 digital literacy tools in eHLA demonstrated such a correlation.

This may reflect that people who perceive their own health to be good are more engaged in information and technology and are more motivated than those with poor self-rated health. This is in contrast to Neter and Brainin (2012) who examined the relation between self-rated health and eHL in an Israeli population study and to Milne et al (2015) who examined the relation between perceived health and eHL in patients with lung cancer. This may be explained by differences in the instrument used to assess the self-reported health as well as measurements of eHL [17,38].

### Limitations of the Study

A limitation of the study is that it is an observational study conducted in an outpatient clinic in a Danish region where people in general have a rather high sociodemographic profile with respect to income and education.

It should also be noted that the data about digital behavior build on self-reported information and not data acquired from the systems. This may introduce a bias.

Another limitation is the mandatory usage of NemID in Denmar, which may have resulted in a selected population for this investigation compared with other countries with a lower degree of digitalization.

Using 2 instruments, each with 7 scales in the evaluation, may have resulted in a multisignificance problem, where we can have obtained type I error. We have accommodated this by applying the Bonferroni correction. On the other hand, this may have introduced a risk of type II error because of insufficient power of the study caused by the sample size [32]. Further studies that are designed to test our findings, with enough power, are necessary before further conclusions can be drawn.

### Conclusions

Our results contribute to the growing knowledge about which factors are important for use of digital health services. Our data show that there were no significant differences between users and nonusers of the digital health service sundhed.dk with regard to age, sex, or educational level. Therefore, these factors alone cannot be used to guide health professionals to understand their patients’ adoption and usage of sundhed.dk. However, significant differences were identified between users and nonusers in almost of all the tools of eHLA and eHLQ dimensions. This supports the notion that skills, motivation, and experience of health and digital services are related to the adoption and usage of technology [6,7].

The results emphasize that multifaceted measurements of eHL may be able to capture the factors important to the adoption of digital health services and thereby serve to guide health professionals to better understand and support their patients to obtain the full benefits of the increasing digitalization of the health care sector.

Further studies are needed to identify how the tools or the underlying dimensions can be best used to inform the clinicians and facilitate that more patients take advantage of digital health services and technologies and benefit from the ever-expanding evolution of digital health.

## Abbreviations

e-Boks: Patient’s digital mailbox
eHEALS: eHealth literacy scale
eHealth: electronic health
eHL: eHealth literacy
eHLA: eHealth literacy assessment
eHLF: eHealth literacy framework
eHLQ: eHealth literacy questionnaire
EQF: European Qualifications Framework
GP: general practitioner
ICT: information and communications technology
ISCED: International Standard Classification of Education
